# The effectiveness of a feminist-informed, individualised counselling intervention for the treatment of eating disorders: a case series study

**DOI:** 10.1186/s40337-022-00592-z

**Published:** 2022-05-18

**Authors:** Jessica Tone, Belinda Chelius, Yvette D. Miller

**Affiliations:** 1 School of Public Health and Social Work, Queensland University of Technology, Victoria Park Road, Kelvin Grove, QLD 4059 Australia; 2 Eating Disorders Queensland, 51 Edmondstone Street, South Brisbane, QLD 4101 Australia

**Keywords:** Feminist therapy, Sociocultural approach to eating disorders, Trauma-informed practice, Community treatment, Integrative psychotherapeutic approach, Outpatient

## Abstract

**Background:**

Currently, there is limited empirical validation of feminist-informed or individualised interventions for the treatment of eating disorders. The aim of this study was to examine the effectiveness of a feminist-informed, individually delivered counselling intervention for the treatment of eating disorders at a community-based eating disorder treatment service.

**Methods:**

Eighty individuals aged between 17 and 64 years presenting to an outpatient eating disorder service were examined in a case series design at baseline, session 10, session 20 and end of treatment (session 30). Changes in eating disorder symptomology, depression, anxiety, stress, and mental health recovery over the course of treatment were examined in linear mixed model analyses.

**Results:**

The treatment intervention was effective in reducing eating disorder symptomology and stress and improving mental health recovery after 10 sessions in a sample of 80 eating disorder participants engaged with the treatment service. Reductions in eating disorder symptomology and stress and improvements to mental health recovery were maintained at session 20 and session 30.

**Conclusions:**

The findings of this study provide preliminary support for feminist-informed and individualised interventions for the treatment of eating disorders in community-based settings.

## Plain English Summary

Eating disorders can result from a variety of factors including previous trauma and sociocultural influences. Critical feminist perspectives acknowledge these influences are core contributing factors to the development and maintenance of eating disorder behaviours and postulate the exploration of the eating disorder in relation to these wider factors as crucial to the treatment process. Therefore, treatment interventions that utilise feminist frameworks and approaches that are integrative of a variety of psychological therapies to suit individual needs may be useful to address underlying factors while also managing eating disorder behaviours. However, there have been few experimental studies that have evaluated these interventions. This article aims to address this gap in current eating disorder literature by describing and evaluating the effectiveness of a counselling therapy for eating disorders that employs feminist practice and a variety of psychological therapies. The results indicate that eating disorder symptoms, stress, and mental health recovery improved after 10 sessions of the counselling intervention for a sample of 80 participants receiving eating disorder treatment. The results from this study provide initial evidence for the usefulness of feminist-informed practice and individualised counselling interventions for the treatment of eating disorders.

## Background

Eating disorders (EDs) are characterised by persistent disturbances in eating-related behaviours and attitudes, often occurring alongside body image concerns and overvaluations of body shape and weight [1]. Eating disorders are increasingly recognised as important causes of morbidity and mortality, associated with medical and psychiatric comorbidities [1, 2], social and physical functional impairment, reduced quality of life [3, 4], and an increased risk of all-cause and suicide mortality [5–7]. The aetiology of ED symptomology is complex and multifaceted, resulting from interactions between sociocultural, environmental, psychological, and biological factors [8, 9]. The clinical presentations, sociocultural and environmental experiences, as well as social demographics of people who experience disordered eating behaviours vary greatly [10, 11]. Moreover, adding to the complexity of EDs, traumatic environmental factors such as physical, sexual, and emotional childhood maltreatment have been associated with ED symptomology, with estimated prevalence of childhood maltreatment of 17–46% [12] and 19% of any traumatic event [13] in ED samples. In this regard, ED behaviours may emerge as maladaptive coping mechanisms to deal with distress caused by traumatic life experiences [14, 15].

### Integrative and individualised treatment approaches

Given the complexity of and variability within EDs, individualised treatment approaches ensure treatment interventions meet the needs of the individual and address both ED behaviours and the causal and maintaining factors [15]. Current practice standards and guidelines emphasise personalised implementation of evidence-based practices [16]. Evidence-based practice refers to the integration of empirically supported research, clinical expertise, and stakeholder perspectives in the context of patient characteristics, culture, and preferences [17, 18]. Empirically supported psychotherapies for EDs include cognitive behavioural therapy (CBT), ED focused enhanced CBT (E-CBT), family-based therapy, and interpersonal psychotherapy (IPT) [16, 19, 20]. There is emerging evidence for the efficacy of other psychotherapies, including dialectical behavioural therapy (DBT) and acceptance and commitment therapy (ACT) [21–23]. The highly manualised psychotherapies implemented in clinical trials are known to be less rigorously implemented in terms of fidelity in community environments [24]. A large proportion of ED community practitioners report implementing integrative psychotherapeutic approaches (i.e., integrating multiple approaches or psychotherapies) [24]. This may reflect the substantial variations in ED patients presenting for treatment, including in ED symptomology, psychiatric comorbidities and the underlying issues contributing to their EDs [11, 25].

Despite their widespread use there is a lack of empirical evidence describing individualised or integrative counselling interventions for community ED treatment and the effectiveness of such approaches. The limited evidence that does assess such approaches has primarily focused on inpatient and intensive day programs. For example, an intensive day program in an American ED treatment centre has been evaluated in three studies [17, 26, 27]. The treatment intervention offered an individualised counselling framework integrating CBT and psychodynamic frameworks with other psychotherapies including ACT, DBT, IPT, gestalt therapy, and body image therapy, completed alongside a multimodal treatment delivery of group therapy, individual therapy, family therapy, and dietetic support. Comparing pre- and post-treatment scores of participants who completed the intensive outpatient day program or partial hospitalisation program, all three studies demonstrated statistically significant decreases in ED symptomology and depression following treatment in three samples of ED patients who on average remained in the program for 13 weeks.

While these studies provide preliminary empirical evidence for the effectiveness of individualised and flexible treatment programs, to our knowledge there remains a lack of studies describing and assessing individualised counselling interventions implemented in less-intensive, community-based facilities. Furthermore, there is limited up-to-date evidence published in the last 5-years, with two of the abovementioned studies published over a decade ago. Evaluating community-based treatment programs will offer insight into the effectiveness of treatment interventions that are currently implemented for the treatment of EDs in widely accessed community treatment centres. This is important to offer insight into how empirically supported, evidence-based practices are operationalised in real-world treatment settings.

### Feminist frameworks for eating disorders

The aetiology of EDs and the factors that maintain ED behaviours need to be considered for the implementation of personalised treatment interventions. Feminist perspectives postulate social, cultural, political, and environmental factors as core aetiological features of EDs [28–31]. These approaches differ from biomedical models in that the development and maintenance of ED symptomology is examined in relation to wider sociocultural influences instead of individual pathology [32]. Theoretical feminist models have considered the influences of cultural discourses of thinness idealisation, family, peer, and media ideologies of bodies, restricted agency in relation to gender experience, experiences of objectification, harassment, or assault, and intersections with political structures of power and oppression related to gender, race, cultural background, sexuality, and class [28, 29, 31, 33–36]. In this regard, EDs are viewed not just as eating disturbances and body image problems, but as complex responses to environmental, sociocultural, and political stressors [30].

There is a growing empirical foundation supporting sociocultural and environmental factors as aetiological features of EDs [12, 13, 29, 31, 37–39]. Feminist and sociocultural perspectives have contributed to the basis of several prevention programs targeted at the sociocultural influences impacting eating behaviours and body image [29, 40]. Nonetheless, a focus on individual pathology remains at the forefront of clinical ED interventions [41]. While a great deal has been written about EDs from feminist and sociocultural perspectives, these frameworks have seen minimal translation into contemporary ED treatment [31, 41]. This may be due to the emphasis placed on the implementation of evidence-based practices [16, 42], and the current lack of empirical validation for the value of feminist frameworks in ED treatment contexts [43].

The integration of feminist frameworks with evidence-based psychotherapies have been proposed in several treatment models [43–45]. These models aim to incorporate key aspects of feminist therapy, including exploration of wider aetiological factors, client empowerment, and promotion of egalitarian therapeutic relationships. Psychotherapies such as CBT, DBT, and IPT are used to develop alternative coping strategies, reduce ED behaviours, and improve interpersonal relationships [43–45]. Anecdotally, the implementation of psychotherapies underpinned by a feminist framework provides a treatment model capable of addressing the complexity of ED presentations that moves away from the dominant biomedical model of individual pathology. However, the effectiveness of these models has not been empirically substantiated. Empirical examination of such a model will offer both critical insight into alternative treatments for EDs and contribute to decreasing the empirical gap between sociocultural and biomedical paradigms [32].

### Aims and objectives

Given the scarcity of empirical examination of both individualised and feminist-informed ED treatment interventions, the aim of the current study was to examine the effectiveness of a feminist-informed and individualised counselling intervention for the treatment of EDs. Eating Disorders Queensland (EDQ) is a state-wide, outpatient ED treatment service located in Brisbane, Australia. Their treatment services are underpinned by a feminist perspective, offering an alternative approach to the biomedical model in a non-clinical, community-based environment. The individual counselling frameworks are inclusive of feminist practice, employing an integrative psychotherapeutic approach and individual tailoring of treatment programs. The primary objective of this study was to evaluate the impact of this service model on the trajectory of ED treatment and recovery outcomes in a clinical sample of participants engaged in ED counselling.

## Methods

### Design and procedure

This retrospective observational case series study was conducted using de-identified participant data collected by EDQ as part of routine practice from July 2018 to May 2021. As part of EDQ’s intake process, all participants who engage with the service give written consent for the use of their de-identified outcome measurements and unidentifiable demographic data for research purposes, including the use of aggregated results in published research. Due to the retrospective nature of the study, participants were not given information specific to this research at the time of consenting. However, all participants who engage with EDQ’s services are given information about the opportunity to modify or withdraw consent at any time. No participants included in this study modified or withdraw their consent over the course of their treatment. Self-report outcome measures were administered by EDQ prior to the initial counselling session (pre-treatment/baseline) and then at approximately 10-session intervals in single-group, longitudinal design. Measures were electronically administered to participants via email and completed in their own time. Outcomes of interest were changes in continuous measures of ED symptomology, common co-occurring negative emotional states (depression, anxiety, stress) [2], and mental health recovery over the course of treatment.

### Treatment intervention

The treatment intervention comprised up to 30 individual counselling sessions completed at an individualised rate according to participants’ needs. The total number of sessions completed also varied between participants and was determined based on a variety of factors such as ED presentation, severity, and underlying factors or trauma. As EDQ is a state-wide service, counselling sessions were carried out either face-to-face or via telehealth (over the telephone or Zoom/Microsoft Teams) for participants who resided outside of the region for accessible face-to-face delivery. Due to the Coronavirus Disease 2019 (COVID-19) pandemic, all treatments were offered via telehealth between March 2020 and August 2020. Counselling interventions were performed by EDQ’s clinical practitioner team, comprising psychologists, counsellors, and social workers.

The counselling intervention was underpinned by a feminist framework, shifting focus from individual pathology to broader sociocultural and environmental influences. This included examining the precipitating and maintaining factors of each participant’s ED through a biopsychosocial lens and tailoring treatment to further explore identified factors in relation to the ED behaviours. In treatment, the traditional focus on numbers regarding body weight and food was shifted to exploring the participant’s experience of an ED, considering social, political, and environmental impacts. Practitioners focused on building a strong therapeutic alliance through key aspects of feminist-informed practice, including client empowerment, providing information for collaborative and informed decision making, clear communication and transparency, co-creating a safe and supportive environment, and reducing power differentials within the therapeutic relationship. In line with both feminist and person-centred practice, each individual participant was placed at the centre of their treatment, with practitioners taking value from the lived experience to recognise the skills, strengths, expertise, and knowledge that individuals bring to their own lives [46].

The course of treatment typically followed an initial focus on the management and intervention of ED behaviours, followed by the identification and exploration of underlying causes and trauma through trauma-informed and feminist frameworks. A range of empirically supported and emerging psychotherapies were integrated into treatment plans to suit individual participant needs and goals in line with evidence-based practice, including CBT, DBT, ACT, narrative therapy, and expressive therapies. The use of psychotherapies varied between individual treatment plans and was based on the clinical judgement of the treating practitioner, the identified precepting and maintaining factors, and the therapeutic needs of individual participants identified through intake assessments and throughout the course of treatment. Practitioners aimed to create opportunities for participants to recognise and validate the negative experiences or trauma that the ED may have assisted in coping with, while supporting participants to implement alternative coping strategies and enhance capacity to seek support within relationships. Participants were also supported to undergo external clinical management of physical symptoms with a general practitioner (GP), through Specialist Supportive Clinical Management (SSCM) to ensure the medical comorbidities that can coincide with EDs were managed. Detailed information about the treatment approach and practice framework is available elsewhere [46, 47].

### Participants

**Fig. 1:**
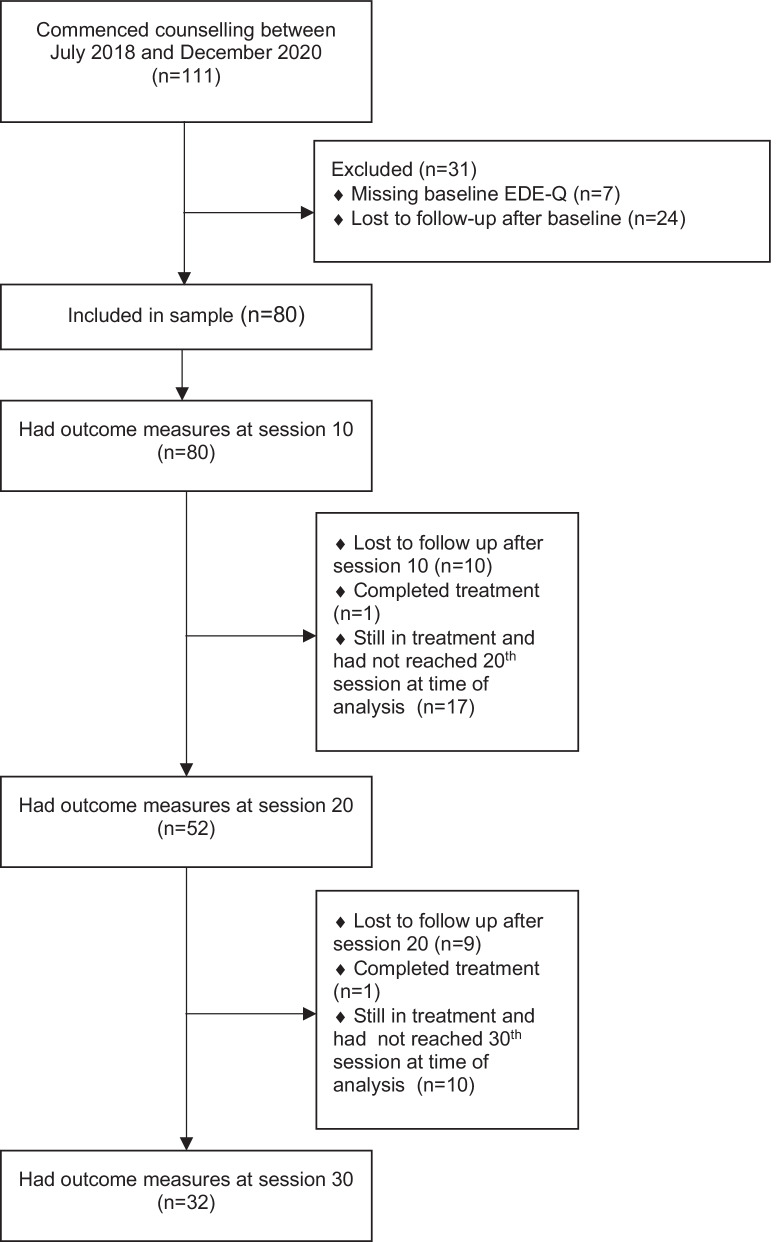
Participant flow through treatment

De-identified scores on outcome measures, age, gender, and dates of measurement completion were provided by EDQ for all participants who attended an initial counselling session at EDQ for an ED or disordered eating between July 2018 and December 2020. Participant data was de-identified by EDQ by removing participant names and other identifying details such as date of birth from the data set. All participants self-referred to the treatment service. A formal ED diagnosis was not required to access treatment services at EDQ nor applied as an inclusion criterion for this study. Participants were not included in the study if they had commenced counselling at EDQ during this period but were determined to be still engaged with EDQ’s individual counselling intervention and had completed less than 10 sessions of treatment by May 2021. All other participants who commenced treatment during this time were assessed against further inclusion criteria of: (1) at least one follow-up measure after baseline and (2) had a baseline measure of ED symptom severity (Eating Disorder Examination Questionnaire [EDE-Q] Global score). Of the 111 participants identified during the first inclusion stage, 80 (72.1%) met further inclusion criteria and had adequate follow-up data for analyses. Figure 1 shows the study sample flow through the treatment service.

### Outcome measures

#### Eating disorder symptomology

The Eating Disorder Examination Questionnaire (EDE-Q) [48, 49] is a self-reported derivative of the Eating Disorder Examination Interview (EDE) [50]. It is a 28-item measure of ED psychopathology commonly used to assess changes in ED symptomology over the course of treatment. Twenty-two of the 28 Items are scored on a 7-point, Likert scale (range = “0” to “6”), with respondents asked to rate the frequency or impact of key ED behaviours and psychological features over the past 28-days. Four subscale scores: Restraint (5 items); Eating Concern (5 items); Shape Concern (8 items); and Weight Concern (5 items; 1 item repeated from Shape Concern), and a global score are derived from these 22 items, ranging between 0 and 6. Higher scores are indicative of greater severity. The subscale and global scores of the EDE-Q have previously been shown to have acceptable internal consistency, discriminant validity, and to be a valid measure of ED symptomology [51, 52]. In this study, Cronbach’s α values at each timepoint for the Restraint, Eating Concern, Shape Concern and Weight Concern subscales and the EDE-Q Global score ranged between 0.81 and 0.90, 0.60–0.75, 0.89–0.92, 0.81–0.86, and 0.85–0.90 respectively. Behavioural frequency items of the EDE-Q were not utilised in the current analysis as they do not contribute to subscale or overall scores.

#### Depression, anxiety, and stress

The Depression Anxiety Stress Scales (DASS-21) is a self-reported measure of negative emotional states. It is a short form of the original 42-item DASS [53] and consists of 21 items that form three, 7-item scales corresponding to depression, anxiety, and stress. Respondents are asked to rate the extent to which they have experienced symptomology of depression, anxiety, and stress over the past 7-days on a 4-point rating scale (range = “0” to “3”). Higher scores indicate greater experiences of symptomology (scale score range = 0 to 42). The DASS has acceptable internal consistency and convergent and discriminate validity and has been demonstrated as a valid measure of routine clinical outcomes [54–56]. In this study, the Cronbach’s α values for each timepoint ranged between 0.90 and 0.95 for the depression scale, 0.80–0.89 for the anxiety scale, and 0.81–0.88 for the stress scale.

#### Mental health recovery

The Recovery Assessment Scale–Domains and Stages (RAS-DS) [57] is a self-reported measure of mental health recovery. It consists of 38 items that are rated on a 4-point Likert scale (range = “1” to “4”). In addition to a total recovery score (range = 38 to 152), the RAS-DS generates four subscales that correspond to different recovery domains: Doing Things I Value (6 items; range = 6 to 24); Looking Forward (18 items; range = 18 to 72); Mastering My Illness (7 items; range = 7 to 28); and Connecting and Belonging (7 items; range = 7 to 28). Higher scores indicate better recovery. The RAS-DS has acceptable internal consistency and construct validity and has been demonstrated to be sensitive to changes in recovery over time [58, 59]. In this study, Cronbach’s α values at each timepoint for the Doing Things I Value, Looking Forward, Mastering My Illness, and Connecting and Belonging subscales and the RAS-DS total score ranged between 0.77 and 0.85, 0.87–0.94, 0.73–0.91, 0.69–0.85, and 0.90–0.97 respectively.

### Missing data

#### *Eating disorder* examination questionnaire

Subscale scores were calculated if the number of missing items for a subscale were not less than half the total number of items corresponding to that subscale, following methods recommended by Fairburn and Cooper [60]. Global scores were calculated when there were at least two of the four subscale scores available.

#### Depression anxiety stress scales

Scale scores on the DASS-21 were calculated if participants had at least six items of the 7-item scale. Where participants had six of the 7-items, the missing item was imputed as the average of the remaining six scale items before the scale score was calculated.

#### Recovery assessment scale–domains and stages

Subscale scores on the RAS-DS were calculated if participants had at least half of the items within a subscale. Where participants were missing only selected items but not more than half of the items on any subscale, missing raw scores were imputed as the average from the available items before the subscale score was calculated, consistent with methods used by Hancock and colleagues [58]. There were no instances where missing items or subscale scores impacted the calculation of the total score.

### Statistical analysis

All analyses were performed using IBM SPSS Statistics (version 27). To determine the effect of the intervention, separate linear mixed models (LMMs) were constructed for each continuous outcome variable. Each model was fitted with fixed effects of time in treatment (ordinal; 1 (referent) = baseline, 2 = session 10, 3 = session 20, 4 = session 30) and the EDE-Q global score at baseline to control for variations in ED symptom severity at baseline (continuous). Random effects were specified in all LMMs with a random intercept for subject and a scaled identity covariance structure. The restricted maximum likelihood (REML) estimation was used for all models. Satterwaite approximation was used to compute degrees of freedom. All main effects were considered statistically significant at an alpha level of *p* < 0.05. To examine outcome trajectory, significant main effects of time in treatment were further examined using pairwise comparisons between timepoints. Pairwise comparisons were considered statistically significant at an alpha of *p* < 0.01 to reduce type I error associated with multiple comparisons. Results are presented as the mean (*M*) change with 99% confidence intervals (99% CI) over the course of treatment.

## Results

### Sample characteristics

The sample included 80 participants who ranged in age at baseline between 17- and 64- years (*M* = 30.24 years, *SD* = 12.29 years). Seventy-three participants identified as female (91.2%) and seven identified as male (8.8%). The average treatment time-period between baseline and session 10 was 117.55 days (*SD* = 59.86 days); between session 10 and session 20 was 122.73 days (*SD* = 55.31); and between session 20 and session 30 was 153.94 days (*SD* = 53.76). After session 10, one participant completed their treatment program (1.3%), 17 participants had not yet reached their 20th session (21.3%), and 10 participants were lost to follow-up (I.e., did not fill out subsequent outcome measures) (12.5%). At session 20, 52 participants in the sample had outcome measures (65%). After session 20, one participant had completed their treatment program (1.3%), 10 participants had not yet reached their 30^th^ session (12.5%), and nine were lost to follow-up (11.3%). Thirty-two participants in the final sample had outcome measures at session 30 (40%) (see Fig. 1).

### Outcome analyses

#### Eating disorder symptomology

Means and standard deviations for all outcomes at each timepoint are described in Table 1. A significant main effect of time in treatment was observed for the EDE-Q global score (*F*(171.26) = 25.65, *p* < 0.001) and four subscales: Restraint (*F*(160.51) = 23.10, *p* < 0.001), Eating Concern (*F*(11.40) = 165.20, *p* < 0.001), Shape Concern (*F*(164.64) = 15.89, *p* < 0.001), and Weight Concern (*F*(163.80) = 11.09, *p* < 0.001), when controlling for ED symptom severity (EDE-Q global score) at baseline. Significant main effects of time in treatment were due to significant reductions in EDE-Q global score and all subscales at session 10, session 20, and session 30, in comparison to baseline (see Table 2). Pairwise comparisons indicated that there were no significant changes between session 10, session 20, and session 30 (across all possible comparisons) for all EDE-Q outcomes (Table 2).

**Table 1:** Means and standard deviations for outcomes over the course of the treatment intervention

	*M (SD)*			
	T_0_ (*n* = 80)	T_1_ (*n* = 80)	T_2_ (*n* = 52)	T_3_ (*n* = 32)
EDE-Q global	3.74 (1.27)	3.05 (1.37)	2.85 (1.42)	2.79 (1.47)
Restraint	3.18 (1.56)	2.24 (1.59)	1.96 (1.64)	1.96 (1.74)
Eating concern	3.13 (1.21)	2.60 (1.42)	2.27 (1.40)	2.15 (1.43)
Shape concern	4.46 (1.53)	3.72 (1.66)	3.77 (1.82)	3.61 (1.80)
Weight concern	4.14 (1.50)	3.55 (1.70)	3.40 (1.72)	3.44 (1.69)
Depression	19.43 (10.15)	16.59 (10.38)	17.04 (11.31)	17.63 (11.78)
Anxiety	16.68 (8.89)	12.38 (8.07)	11.50 (8.21)	13.63 (9.93)
Stress	23.57 (8.47)	19.70 (7.74)	19.46 (9.18)	19.63 (9.25)
RAS-DS Total	100.33 (14.74)	107.52 (16.75)	108.71 (19.31)	122.32 (22.01)
Doing things I value	17.35 (3.20)	17.99 (3.11)	18.12 (3.41)	18.29 (3.93)
Looking forward	46.46 (8.16)	49.64 (9.02)	49.55 (9.98)	50.87 (11.35)
Mastering my illness	15.71 (3.45)	18.39 (4.30)	19.22 (4.31)	20.77 (4.59)
Connecting and belonging	20.81 (4.24)	21.56 (3.85)	21.82 (4.84)	22.39 (4.88)

*EDE-Q* eating disorder examination questionnaire, *RAS-DS* recovery assessment scale–domains and stages

**Table 2:** Mean change over the course of the treatment intervention

Outcome	*M* change^a^ (99% CI)				
	T_0_–T_1_	T_1_–T_2_	Total change T_0_–T_2_	T_2_–T_3_	Total change T_0_–T_3_
EDE-Q global	− 0.68 [− 0.98, − 0.38]*	− 0.29 [− 0.64, 0.07]	− 0.96 [− 1.31, − 0.62]*	− 0.09 [− 0.53, 0.35]	− 1.05 [− 1.47, − 0.64]*
Restraint	− 0.90 [− 1.31, − 0.50]*	− 0.37 [− 0.85, 0.11]	− 1.27 [− 1.74, − 0.80]*	− 0.03 [− 0.62, 0.57]	− 1.30 [− 1.86. − 0.74]*
Eating concern	− 0.48 [− 0.88, − 0.08]*	− 0.36 [− 0.82. 0.10]	− 0.84 [− 1.30, − 0.39]*	− 0.16 [− 0.74, 0.42]	− 1.00 [− 1.54, − 0.45]*
Shape concern	− 0.71 [− 1.06, − 0.36]*	− 0.09 [− 0.50, 0.33]	− 0.80 [− 1.20, − 0.40]*	− 0.18 [− 0.70, 0.33]	− 0.98 [− 1.47, − 0.50]*
Weight concern	− 0.57 [− 0.96, − 0.19]*	− 0.26 [− 0.71, 0.19]	− 0.83 [− 1.27, − 0.39]*	− 0.03 [− 0.6, − 0.53]	− 0.86 [− 1.40, − 0.33]*
Depression	− 2.63 [− 5.54, 0.28]	− 0.41 [− 3.81, 2.99]	− 3.04 [− 6.45, 0.37]	0.63 [− 3.53, 4.78]	− 2.41 [− 6.40, 1.58]
Anxiety	− 3.83 [− 5.97, − 1.68]*	− 0.95 [− 3.45, 1.55]	− 4.78 [− 7.29, − 2.26]*	2.17 [− 0.88, 5.22]	− 2.61 [− 5.55, 0.34]
Stress	− 3.57 [− 6.10, − 1.05]*	− 0.31 [− 3.24, 2.62]	− 3.88 [− 6.82, − 0.94]*	− 0.20 [− 3.79, 3.40]	− 4.08 [− 7.51, − 0.64]*
RAS-DS Total	6.23 [2.01, 10.44]*	1.83 [− 3.11, 6.78]	8.06 [3.25, 12.86]*	4.03 [− 2.01, 10.07]	12.09 [6.35, 17.83]*
Doing Things I Value	0.36 [− 0.47, 1.20]	0.23 [− 0.75, 1.20]	0.59 [− 0.36, 1.54]	0.17 [− 1.02, 1.36]	0.76 [− 0.37, 1.89]
Looking Forward	2.69 [0.29, 5.09]*	0.64 [− 2.17, 3.45]	3.33 [0.59. 6.06]*	1.38 [− 2.06, 4.82]	4.71 [1.44, 7.97]*
Mastering My Illness	2.72 [1.49, 3.94]*	0.71 [− 0.72, 2.14]	3.34 [2.04, 4.82]*	1.67 [− 0.08, 3.42]	5.10 [3.44, 6.75]*
Connecting and Belonging	0.49 [− 0.42, 1.40]	0.22 [− 0.85, 1.29]	0.71 [− 0.34, 1.76]	0.83 [− 0.48, 2.15]	1.54 [0.29, 2.80]*

*EDE-Q* eating disorder examination questionnaire, *RAS-DS* recovery assessment scale–domains and stages

*p < .01

^a^Mean change adjusting for ED severity at baseline as estimated by linear mixed models

#### Depression, anxiety, and stress

A significant main effect of time in treatment was observed for anxiety (*F*(151.41) = 10.76, *p* < 0.001) and stress (*F*(159.40) = 6.56, *p* < 0.001), but not for depression (*F*(153.68) = 2.60, *p* = 0.054), when controlling for ED symptom severity at baseline. Significant main effects of time in treatment on anxiety were due to significant reductions at session 10 and session 20 in comparison to baseline. However, the mean anxiety score at session 30 was not significantly different in comparison to baseline scores, indicating a J-shaped trend between baseline and session 30 (Table 2). Significant main effects of time in treatment on stress were due to significant reductions at session 10, session 20, and session 30, in comparisons to baseline. Pairwise comparisons indicated that there were no significant changes in anxiety or stress between session 10, session 20, and session 30 (across all possible comparisons) (Table 2).

#### Mental health recovery

A significant main effect of time in treatment was observed for the RAS-DS total score (*F*(151.97) = 12.89, *p* < 0.001) and subscales: Looking Forward (*F*(153.07) = 6.46, *p* < 0.001), Mastering My Illness (*F*(156.54) = 27.89, *p* < 0.001), and Connecting and Belonging (*F*(149.55) = 3.59, *p* = 0.015), but not for the Doing Things I Value subscale (*F*(151.23) = 1.42, *p* = 0.238), when controlling for ED symptom severity at baseline. Significant main effects of time in treatment on the RAS-DS total score and Looking Forward and Mastering My Illness subscales were due to significant increases at session 10, session 20, and session 30, in comparison to baseline. Pairwise comparisons indicated that there were no significant changes between session 10, session 20, and session 30 (across all possible comparisons) on both the RAS-DS total score and Looking Forward subscale (Table 2). However, a further significant mean increase of 2.38 (99% CI: 0.70, 4.06, *p* < 0.001) on the Mastering My Illness subscale was observed between session 10 and session 30. There were no significant changes between session 10 and session 20, nor between session 20 and session 30 on the Mastering My Illness subscale. Significant main effects of time in treatment on the Connecting and Belonging subscale were due to significant increases at session 30 in comparison to baseline. There were no other significant changes observed between timepoints on the Connecting and Belonging subscale (Table 2).

## Discussion

This study aimed to describe the effectiveness of a feminist-informed and individualised counselling intervention for EDs, delivered in an outpatient community-based setting. The results indicated that for the current sample, an individualised counselling intervention underpinned by a feminist framework may be effective in reducing ED symptomology within the first 10 sessions, over approximately 18 weeks. The greatest change in outcomes occurred during the first 10 sessions of treatment. The reductions observed in the ED symptomology during the initial stages of treatment are promising, with previous evidence suggesting that early response to ED treatment is associated with better ED symptomology outcomes [61]. There were no further significant changes observed beyond session 10 for ED symptomology outcomes in this study. However, improvements observed during the initial 10 sessions were maintained at later timepoints, indicating a significant reduction in ED symptomology at the conclusion of treatment. The reductions in anxiety observed at session 10 were maintained at session 20. However, improvements in anxiety were not maintained further, with mean anxiety scores over the full course of the treatment intervention following a J-shaped trend. We observed no change in depression over the course of treatment. Future consideration should be made to include additional follow-up timepoints to explore long-term treatment trajectories in relation to the observed early improvements to ED symptomology and to explore the long-term course of the J-shape trend we observed in anxiety scores.

Overall mental health recovery improved within 10 sessions. There were further improvements observed between session 10 and session 30 on the Mastering My Illness RAS-DS subscale, which reflects the patient’s sense of “control over, or management of, any residual symptoms” [57, p.7]. The Connecting and Belonging RAS-DS subscale, measuring interpersonal relationships, social functioning, and societal participation, was not observed to change until session 30. Both results suggest that the full length of the treatment intervention, which allocated a larger focus on addressing the underlying factors or trauma contributing the ED, was important for improving aspects of recovery-orientated change. Eating disorder recovery should not only defined by the reduction of ED signs and symptoms [62]. Feminist-informed practice places emphasis on shifting the focus from signs and symptoms to patient experience [29]. Thus, exploration of recovery-orientated measures (e.g., the RAS-DS) are important when considering the effectiveness of ED treatment interventions and the value of feminist frameworks. The inclusion of a recovery-orientated measure in the present study extends on previous evaluation of individualised ED treatment interventions [17, 26, 27] that have solely focused on outcomes of ED and psychiatric symptom measurement, by demonstrating the value of an integrative, feminist-informed intervention for both reducing ED symptomology and improving features of mental health recovery. Supplementary qualitative outcome measures in future research would significantly add to the evaluation of feminist-informed ED treatment in assessing recovery-orientated impacts.

The observed reductions in ED symptomology are broadly comparable to previous studies by Schaffner and colleagues [17, 26] and Freudenberg and colleagues [27] reporting the effects of individual, multimodal treatment interventions implemented in observation studies, in that ED symptomology was observed to reduce from pre-test to post-test. However, the delivery of the current intervention was less intensive at approximately one session per fortnight, in comparison to a minimum of one session per week and up to three sessions per day in previous studies [17, 26, 27]. It is important to evaluate treatment interventions outside of intensive or rigid inpatient treatment settings to ensure effective treatment is accessible to individuals who are both exiting tertiary treatment facilities and to prevent deterioration of less severe ED presentations in the community. Providing effective, ED specific community-based treatment is crucial to the Australian National Stepped Care approach, which outlines a continuum of care to ensure individuals can step up or step down the intensity of their treatment based on their current needs [63, 64].

To our knowledge, this is the first study to evaluate the effectiveness of a feminist-informed, individual treatment intervention integrated with empirically supported psychotherapies for the reduction of ED symptomology. Theoretical feminist literature has significantly contributed to the biopsychosocial model of EDs, postulating sociocultural and relational factors as core etiological features to begin a pivotal shift in the way we think about EDs [65]. Despite this, feminist-based therapy is largely excluded from consideration as an evidence-based treatment for EDs [31, 41]. The integration of feminist therapy through the exploration of wider sociocultural aetiological factors with psychotherapies to reduce ED behaviours and thoughts has been previously described in several treatment models [43–45]. The results of this study provide preliminary evidence for such models when used in community-based ED treatment settings, contributing to empirical validation of feminist treatment approaches. Ongoing evaluation of feminist treatment approaches for EDs is warranted to further substantiate this preliminary evidence and highlight the value of feminist-based perspectives in ED treatment.

The present study utilised an observational design, analysing the outcome data of participants who had undertaken treatment outside of a research setting. Effectiveness studies undertaken in systematic and dynamic environments are crucial in ensuring efficacy can be translated into real-world practice. Consequently, the preliminary findings of this study provide a basis to further substantiate ecological validity through additional evaluation. In terms of feasibility, the implementation of the intervention in a community ED treatment centre indicates that the intervention should be adaptable to other ED treatment settings. The examination of outcome trajectory allowed for identification of trends that may have otherwise been missed in a pre- to post-treatment comparison. The overall attrition rate and selection bias were also reduced by including all participants with at least one follow-up observation in outcome analyses through LMMs.

The present study is limited by an inability to compare the trajectory of outcomes with a comparison group. Future considerations should be given to comparing the treatment evaluated here against alternative treatment groups to estimate its relative effectiveness. Additionally, it remains unknown whether the 24% of clients in the final sample who were lost to follow-up completed treatment at the last recorded timepoint, completed subsequent sessions but did not complete final measures, or disengaged with the treatment service. This loss to follow up may, in part, have resulted from the data collection procedures, which required clients to complete their final outcome measures after their treatment had concluded. Consequently, it is unknown whether the clients who were lost to follow-up differed in their final outcomes of ED symptomology, psychiatric comorbidities, or recovery, and whether this impacted the completion of their final measures. Further examination into the treatment trajectory of clients who were lost to follow-up and factors related to compliance in completing measures is worth examining in future studies.

Participant characteristics could not be determined beyond age and gender identity. The clinical presentations or diagnoses of the participants and their concomitant exposure to SSCM and other additional treatment services were unknown. Identifying these characteristics in future evaluations would not only improve the generalisability of the results but allow the determination of treatment effectiveness across various ED subtypes (e.g., anorexia nervosa, bulimia nervosa, binge eating disorder) and with or without adjunct therapies. We were unable to examine differences in outcomes between treating practitioners, as the data collected on treating therapist was impacted by a data collection error. All practitioners who provided treatment in the current intervention practice under feminist and trauma-informed frameworks. Exploration of variations in treatment outcomes between different therapists should be explored in future research. Eating Disorders Queensland additionally offers subsequent treatments to individual counselling, including a variety of group therapies. Future considerations should be given to the differences between participants who engage in subsequent services and those who do not, particularly with respect to maintaining the improvements achieved, continued improvements after treatment, and incidence of relapse.

## Conclusions

In all, individualised treatment that integrates a range of psychotherapies with a feminist framework seems to be beneficial for reducing ED symptomology and improving various features of mental health recovery when implemented in an outpatient, community-based setting. A treatment dose of 10 sessions was adequate for the reduction of ED symptomology and stress and improvement of overall mental health recovery. The present study contributes to the small body of research that supports integrative and individualised interventions as a valid intervention for the treatment of EDs. The results of this study additionally provide preliminary support for feminist-informed ED treatments, an area that is currently lacking empirical substantiation. It is crucial to continue evaluation and expansion of community-based ED treatment services to reduce ED presentations to tertiary level treatment facilities and ensure adequate treatment is available to the full spectrum of ED symptom severity. Future studies should include an additional, long-term follow-up period to further substantiate the value of integrative and feminist-informed interventions for sustained ED recovery.

## Data Availability

The de-identified data supporting the results of this study may be available on reasonable request to the corresponding author, subject to agreement from EDQ as the data custodian.

## Abbreviations

ED: Eating disorder
CBT: Cognitive behavioural therapy
E-CBT: Enhanced cognitive behavioural therapy
IPT: Interpersonal psychotherapy
DBT: Dialectical behavioural therapy
ACT: Acceptance and commitment therapy
EDQ: Eating disorders queensland
COVID-19: Coronavirus disease 2019
SSCM: Specialist supportive clinical management
EDE-Q: The eating disorders examination questionnaire
EDE: Eating disorder examination interview
DASS-21: Depression anxiety stress scales
RAS-DS: Recovery assessment scale–domains and stages
LMM: Linear mixed model
REML: Restricted maximum likelihood
df: Degrees of freedom
